# Both High Cognitive Load and Transcranial Direct Current Stimulation Over the Right Inferior Frontal Cortex Make Truth and Lie Responses More Similar

**DOI:** 10.3389/fpsyg.2020.00776

**Published:** 2020-05-19

**Authors:** Nuria Sánchez, Jaume Masip, Carlos J. Gómez-Ariza

**Affiliations:** ^1^Department of Social Psychology and Anthropology, University of Salamanca, Salamanca, Spain; ^2^Department of Psychology, University of Jaén, Jaén, Spain

**Keywords:** transcranial direct current stimulation, deception, cognitive load, inhibitory control, inferior frontal cortex

## Abstract

Deception scholars have argued that increasing the liar’s cognitive system artificially can produce deception cues. However, if too much load is imposed, the truth tellers’ performance can also be impaired. To address this issue, we designed a veracity task that incorporated a secondary task to increase cognitive load gradually. Also, because deception has been associated with activity in the inferior frontal cortex (IFC), we examined the influence of transcranial direct current stimulation (tDCS) of the IFC on performance. During stimulation, participants truthfully or deceptively indicated whether each of a number of statements shown on screen was true or not. Higher load decreased recall but not general compliance or response times (RTs). Truthful trials yielded higher compliance rates and faster RTs than deceptive trials except for the highest load level. Anodal right stimulation decreased compliance in truthful trials when participants were not overloaded. Truth telling was more vulnerable to cognitive load and tDCS than lying.

## Introduction

### Deception and Cognitive Load

Lying has been traditionally considered cognitively more taxing than telling the truth. An early deception detection review suggested that, because of the cognitive difficulty of lying (compared to truth telling), liars might display behavioral signs of cognitive load (Zuckerman et al., 1981)^1^. More recently, Gombos (2006) stressed the relevance of executive functioning to lying, arguing that the liar must inhibit the memory of the truth to deliberately replace it with plausible alternatives. Also, deceivers need to monitor the reactions of the audience, as well as to control their own behavior to appear honest, with all of these activities consuming cognitive resources (Gombos, 2006; see also Vrij et al., 2008a, 2010). Sporer and Schwandt (2006, 2007; Sporer, 2016) additionally stressed the need to take working memory models and research on autobiographical memory into account to understand the cognitive difficulty of lying.

Recent theorizing on the cognitive dimensions of deception considers additional components of executive control besides working memory (Miyake et al., 2000; see also Miyake and Friedman, 2012). Specifically, inhibitory control (the ability to withdraw a predominant response) and task switching (the ability to shift attention between tasks at minimum performance expense) might play a role in deception. Indeed, lying requires keeping the truth in mind while elaborating a deceptive response (working memory), withholding the truth (inhibitory control), and switching between truthful and deceptive responses (task switching) (e.g., Johnson et al., 2004; Spence et al., 2004; Langleben et al., 2005). An example of a recent model considering these elements is Walczyk et al. (2014) Activation–Decision–Construction–Action Theory (ADCAT), according to which the truth is automatically activated in working memory, inhibitory control permits holding it back, and then the lie is made up and released (Walczyk et al., 2014).

Zuckerman et al.’s (1981) contention that the increased cognitive effort involved in lying would naturally result in liars displaying behavioral cues of cognitive load (such as lower speech rate, pupil dilation, or a decrease in body movements) has not been supported. Major meta-analyses have revealed that behavioral indicators associated with deception are rare (DePaulo et al., 2003; Sporer and Schwandt, 2006, 2007) and that it is extremely hard to tell whether someone is lying by observing behavior alone (Bond and DePaulo, 2006). In view of these findings, Vrij et al. (2008a) reasoned that to effectively detect deception, the detector should artificially increase the senders’ cognitive load further (i.e., by asking them to tell their story in reverse order). Under these circumstances, liars (whose cognitive system is already taxed because of lying) will have less cognitive resources left than truth tellers to cope with the increased demands. As a result, they will experience cognitive overload and will show behavioral indicators.

Based on these notions, a number of studies have been conducted recently in which honest and deceptive participants have been interviewed under high or low cognitive load. Two recent systematic reviews showed that imposing load (a) increases behavioral differences between liars and truth tellers relative to a control condition (Vrij et al., 2016) and (b) increases observers’ accuracy in judging veracity from behavior [Vrij et al., 2017; but see also Levine et al.’s (2018) critique]. The effect of imposing cognitive load has also been examined with more artificial laboratory paradigms measuring reaction times (Verschuere et al., 2018).

One way to increase cognitive load is with a secondary task. Not only can liars doing a secondary task display behavioral cues of load; they can also show poorer performance than truth tellers on the task (Vrij et al., 2008b). This effect was demonstrated in a study conducted by Lancaster et al. (2013). Truthful and deceptive participants were requested to perform a haptic sorting task while being interviewed. The results showed that deceivers sorted incorrectly significantly more pieces (per minute) than truth tellers.

In most prior studies, load has been manipulated dichotomously—it is either induced or not (see Vrij et al., 2016, 2017). This might be problematic, as the level of imposed load can be too high, such that even truth tellers are left with little cognitive resources and can show the same indicators of overload as liars (see Blandón-Gitlin et al., 2014; Verschuere et al., 2018). This problem might have serious consequences if cognitive-load approaches to detect deception are to be used in real-world, high-stake settings. For instance, during criminal investigations, innocent suspects displaying signs of cognitive overload could be misclassified as liars. An alternative approach could involve regulating the *degree* of cognitive load being induced, such that the individual’s performance could be measured across a range of increasing load. Using such a parametric manipulation (i.e., with a secondary task of increasing difficulty), the specific amount of cognitive load impairing the liars’ performance on a secondary task but still not hampering the truth tellers’ performance could be empirically determined. Hence, the main goal of this research was to develop a task allowing us to increase cognitive load in a progressive manner. To the extent to which lying is specifically sensitive to cognitive load, gradually increasing cognitive load should make deceptive responses progressively more difficult (relative to truth telling).

### Deception, Executive Control, and Lateral Prefrontal Cortex

A secondary goal of this study was to examine whether neuromodulation of the inferior frontal cortex [IFC; a subregion within the lateral prefrontal cortex (LPFC)] has an effect on several deceptive responses. The rationale behind this purpose is as follows: (a) As discussed above, lying recruits executive control processes; in particular, working memory and inhibitory control processes seem to play important roles in deception. (b) As described below, the IFC has been associated with working memory, inhibitory control, and lying. (c) Therefore, disrupting neural activity of the IFC could be expected to affect responses associated with deception.

The idea that deception strongly recruits (executive) control processes is consistent with the results of neuroimaging studies showing that activity in the LPFC, as well as in other related brain regions (i.e., anterior cingulate cortex), underpins the ability to lie (Gombos, 2006). Indeed, a number of LPFC subregions have been systematically linked to executive functions (i.e., Funahashi, 2001; Miller and Cohen, 2001; Badre, 2008; Dosenbach et al., 2008). In a meta-analysis focused on the contributions of LPFC and executive control to deception, Christ et al. (2009; see also Farah et al., 2014) found that most deception-related prefrontal subregions (10 out of 13) were cortical sites typically associated with working memory, inhibitory control, and/or task switching, with the bilateral IFC concentrating a great deal of activity. For the present study’s purpose, we focus on working memory and inhibitory control constructs.

#### Working Memory

While verbal working memory has been shown to involve a (predominantly left-lateralized) large-scale network (D’Esposito et al., 1998; Chein et al., 2003), the key role of the prefrontal cortex is widely recognized, even though the functional role of these regions remains controversial. Indeed, results of a number of studies using different methodologies (i.e., brain-damaged patients, neuroimaging, neuromodulation) support the idea that dorsolateral prefrontal cortex (DLPFC) importantly contributes to working memory. Thus, for example, in a study with patients, Barbey et al. (2013) found that lesions in the left DLPFC impaired performance in tasks that required the manipulation of information in working memory, whereas damages in the right hemisphere led to deficits in reasoning tasks. Also, a recent meta-analysis concluded that anodal transcranial direct current stimulation (tDCS) over the left (but not the right) DLPFC boosted the benefits of working memory training (Mancuso et al., 2016). The left IFC has been much less studied in relation to working memory in comparison to more dorsal frontal regions, but findings suggest that it might play a role in subvocal rehearsal (Baldo and Dronkers, 2006) as well as in monitoring and scanning working memory contents (Öztekin et al., 2009).

#### Inhibitory Control

The involvement of the right IFC in inhibitory control is now well established, with a number of studies supporting the idea that it is part of a largely right-lateralized network that is involved in motor, memory, and emotional regulation (Anderson and Hanslmayr, 2014; Engen and Anderson, 2018; Castiglione et al., 2019). Thus, for example, using the stop-signal paradigm, which requires one to suppress an ongoing prepotent motor response, studies with patients, neuroimaging, and neuromodulation have revealed a critical role of the right IFC in motor inhibition (for a review, see Aron et al., 2014). Enhanced activity in the right dorso- and ventrolateral prefrontal cortex has also been shown to correlate with reduced activity in the hippocampus, as well as with forgetting, which may be understood as an aftereffect of memory inhibition (for a review, see Anderson and Hanslmayr, 2014). Supporting this interpretation, recent results from neuromodulation studies suggest that cathodal transcranial stimulation over the right LPFC disrupts inhibitory control (Silas and Brandt, 2016; Valle et al., 2020).

#### Brain Stimulation and Deceptive Responding

While neuroimaging studies only provide correlative evidence, non-invasive brain stimulation techniques (such as tDCS) allow researchers to test causal hypotheses on the neural basis of cognitive functions (Filmer et al., 2014; Bestmann et al., 2015; Fertonani and Miniussi, 2016). Specifically, tDCS involves the delivery of a constant weak current (usually 1–2 mA) through two (or more) surface electrodes with at least one of them placed on the participant’s scalp (over the region of interest). The electrical current, which flows from the anodal electrode to the cathodal electrode over a variable period of time (i.e., 20 min), is thought to modulate cortical excitability in the stimulated region and in anatomically connected regions (Romero Lauro et al., 2014). One proposed mechanism that is not completely established is that at a cellular level, anodal stimulation increases neuronal excitability, whereas cathodal tDCS produces the opposite effect (via hyperpolarization) (Liebetanz et al., 2002). Although the specific behavioral effects of stimulating prefrontal areas are still difficult to predict (Jacobson et al., 2012), tDCS is thought to be a useful technique to better understand the involvement of certain brain areas (and networks) in a specific cognitive function (Filmer et al., 2014; Bestmann et al., 2015; Fertonani and Miniussi, 2016). In the present study, we used tDCS of lateral prefrontal regions (specifically the IFC) to investigate its effect on deceptive behavior.

So far only a few studies have used tDCS to investigate deceptive responding, with contradictory results (Priori et al., 2008; Karim et al., 2010; Mameli et al., 2010; Fecteau et al., 2013; Maréchal et al., 2017; Noguchi and Oizumi, 2017). Recently, Bell and DeWall (2018) conducted a meta-analysis on the effectiveness of tDCS to the prefrontal cortex to decrease undesirable social behaviors, including deception. Across the four deception studies they included, effect size was close to zero (Cohen’s *d* = −0.06).

However, the conflicting results might be a consequence of the studies varying greatly in terms of the deception topic (i.e., autobiographical information, general-knowledge information), the brain region stimulated (anterior prefrontal cortex or DLPFC) and laterality (unilateral vs. bilateral stimulation), the stimulation dose or intensity (ranging from 1 to 2 mA), the duration of the stimulation (from 10 to 30 min), whether the data were collected online (i.e., while the participants were being stimulated) or offline (i.e., shortly after the cessation of the stimulation), and the dependent measures used in each study [e.g., while most studies measured behavioral responses such as reaction times, Noguchi and Oizumi (2017) measured observers’ accuracy in judging the participants’ veracity]. This large heterogeneity in terms of procedures and measures, coupled with the scarcity of studies, makes it difficult to draw any firm conclusions. It is thus necessary to conduct additional research on the topic.

In an attempt to pursue this goal, we developed a new task to regulate the amount of cognitive load (operationalized as the number of words to be maintained in working memory) in order to identify how much load is necessary to differentiate between truth telling and lying. In addition, based on previous literature showing that (a) the left IFC plays a crucial role in verbal working memory (e.g., Bunge et al., 2000; Veltman et al., 2003; Owen et al., 2005), (b) the right IFC largely underpins inhibitory control (e.g., Anderson and Hanslmayr, 2014; Aron et al., 2014), and (c) there is a large overlap between deception-related and executive control brain activity (Christ et al., 2009), we used tDCS to modulate neural activity in the IFC to investigate its potential effect on participants’ behavior while performing the truth/lie task. To our knowledge, the present study is the first attempt to modulate deceptive behavior by stimulating the IFC.

### The Current Study

In this study, participants had to indicate truthfully or deceptively whether a number of general-knowledge statements (presented as short sentences) were true or false (for similar procedures, see Gödert et al., 2001; Mameli et al., 2010). We included a secondary task consisting of memorizing the last word in each sentence. Cognitive load was manipulated by progressively increasing the number of sentences presented in each block, and hence the number of words to be memorized by the participants. It is important to note that this procedure largely matches the standard one of reading span tasks that are widely used to assess verbal working memory capacity (i.e., Daneman and Carpenter, 1980; Turner and Engle, 1989), which is usually operationalized as the size of the largest set at which participants reliably recall all of the final words (working memory span). Therefore, our experimental task essentially conformed a reading span procedure, whereby the primary task required subjects to give “yes” or “no” answers to questions involving simple lies or truths, while maintaining an increasing number of words in working memory. During the experimental session, either the left or the right IFC was stimulated with either anodal or cathodal tDCS, so that the experimental design included four separate stimulation conditions (depending on hemisphere and polarity) and a sham condition. We measured three dependent variables: recall (i.e., the percentage of words the participants recalled after each block; see below), compliance (i.e., the extent to which participants were able to follow the instructions to lie or tell the truth), and response time (RT; i.e., the time it took for participants to reply truthfully or deceptively).

### Hypotheses

As for the cognitive load manipulation, we made the following predictions (to be tested in the sham group):

*Hypothesis 1*: Increasing the participants’ cognitive load will lead to decreases in recall.
*Hypothesis 2*: Deceptive responses will be less compliant than truthful responses.
*Hypothesis 3*: Deceptive responses will be preceded by longer RTs than truthful responses.

In addition to these main effects, we also predicted a cognitive load × veracity interaction on compliance and RT. Specifically, and as the main hypotheses of the present study, we expected increases in cognitive load to intensify the truth/lie differences predicted in Hypotheses 2 and 3. Thus:

*Hypothesis 4*: The higher the cognitive load, the larger the difference in compliance between truthful and deceptive responses (predicted in Hypothesis 2) will become.
*Hypothesis 5*: The higher the cognitive load, the larger the difference in RT between truthful and deceptive responses (predicted in Hypothesis 3) will become.

Regarding the impact of tDCS on deceptive responses, the scarcity of relevant knowledge prevented us from posing specific, clear-cut directional hypotheses. Yet, we reasoned that if the IFC plays a role in deceptive behavior (presumably the left IFC being more involved in working memory and the right IFC being more involved in inhibitory control), tDCS of IFC should modulate deceptive behavior relative to sham. However, while we refrained from making any directional prediction, we speculated that tDCS over the left IFC could make compliance more vulnerable to load increases, whereas tDCS of the right IFC could change the putative impact of cognitive load on those conditions that presumably rely heavily on inhibitory control (i.e., deceptive trials). Importantly, to take individual differences in working memory into account, we examined the effects of tDCS on deceptive behavior separately for those cognitive load levels within each participant’s memory span (efficient condition) and those levels surpassing the individuals’ memory span (overloaded condition). We were agnostic about the direction of the putative effects of polarity over our regions of interests [see Bestmann et al. (2015) for arguments in support of this approach] because of the demonstrated non-linearity of the induced effects (anodal tDCS does not necessarily produce enhanced performance, nor does cathodal tDCS always lead to impairments in performance; see Fertonani and Miniussi, 2016). In any case, we expected to contribute to the growing literature on cognition and deception by examining the differential effects of tDCS during lying relative to truth telling.

## Materials and Methods

### Participants

Data were collected from 120 college students at the University of Jaén, Spain. The sample size (*n* = 24 participants in each stimulation condition) was decided prior to conducting the experiment on the basis of the sizes of previous studies on tDCS and deception. Since the number of participants per group in these studies ranged from 6 to 22 (with an average of *n* = 15.50), we decided to have 24 participants per group to approach those experiments with the largest sample size. Participants did not meet any of the following exclusion criteria: history of neurological or psychiatric disorder, drug abuse, susceptibility to seizures, migraines, regular medication, implants, or neurosurgery. However, some participants were excluded because they did not follow the instructions, they did not understand the task, or their values were extreme (see section “Results”). In the end, data from 113 participants were analyzed (90 females; age: *M* = 20.19 years, *SD* = 2.78). The number of participants in each stimulation condition ranged between 21 and 24 (see second column in Table 1). All participants were right-handed according to the Edinburgh Inventory (Oldfield, 1971), and were naïve to brain stimulation. They took part in the experiment voluntarily and received class credit for their participation. The study was approved and carried out in accordance with the recommendations of the Research Ethics Committee of the University of Jaén. All participants were given information about the study and gave written informed consent in accordance with the Declaration of Helsinki.

**TABLE 1:** Sample characteristics.

			Age			Digit Span	
Stimulation							
Condition	*N*	*n*_females_	*M*	*SD*	Range	*M*	*SD*
A-F7	24	19	20.75	3.89	17–37	6.04	1.49
A-F8	22	18	20.14	2.57	17–29	6.14	1.32
C-F7	22	17	20.59	3.19	17–30	6.27	1.98
C-F8	21	17	19.38	1.56	18–25	5.62	2.06
Sham	24	19	20.04	1.97	17–24	6.04	1.40

*A-F7, anode over F7–FC5 and extracephalic cathode; A-F8, anode over F8–FC6 and extracephalic cathode; C-F7, cathode over F7–FC5 and extracephalic anode; C-F8, cathode over F8–FC6 and extracephalic anode.*

### Materials

To create the experimental task, which would require participants to comply with instructions either to lie or not while maintaining words in working memory, we borrowed the structure of Daneman and Carpenter’s (1980) Reading Span Test, and its Spanish-language adaptation (Elosúa et al., 1996). More specifically, we first created a pool of general-knowledge sentences to be answered “yes” or “no” from which we finally selected the 56 experimental sentences. They all were based on the sentences used by Mameli et al. (2010), the Prueba de Amplitud Lectora para niños (PAL-N), which is the spanish translation of reading span test for children (Carriedo and Rucián, 2009), Oswald et al.’s (2015) Reading Span Task, the Information subscale of several Wechsler Adult Intelligence Scale (WAIS) versions (R, III, IV), and the Cervantes Institute’s (2016) test, which measures knowledge about the Spanish constitution and the social and cultural reality of Spain^2^. Sentence length was adjusted such that it ranged from 10 to 13 words and from 19 to 27 syllables^3^. Also, the last word in each sentence had either two or three syllables and was among the most frequent 10,000 Spanish words according to the latest version of the *Corpus de referencia del español actual* (Reference corpus of current Spanish) (Real Academia Española, n.d.).

To make sure that no sentence had double meaning and that the replies were unanimous, in a previous pilot study, we asked 10 young adults to indicate whether each statement (sentence) was true or false, as well as to assess the difficulty to reply to each sentence on a 1-to-5 Likert scale. The sentences were modified until they all had a mean score of 2 on the difficulty scale and all the pilot participants’ replies were accurate.

We also made sure that the last word in each sentence had no connection at all with the last word in the next sentence. To decide which sentences could not go next to each other, we considered (a) free association norms in Spanish (Fernández et al., 2012); (b) semantic categories; (c) graphical, structural, or formal aspects; and (d) other cultural or social aspects.

### Procedure

**FIGURE 1:**
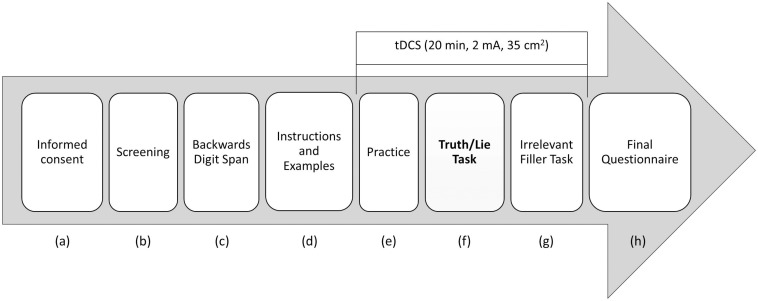
Schematic representation of the experimental procedure.

The full experimental procedure is displayed in Figure 1. The participants came to the laboratory individually. After (a) signing the informed consent form, they (b) completed the screening questionnaire (for us to check whether they were eligible to participate), followed by (c) the backward digit span test, which is a standard measure of memory span. Next, the participants (d) were given the instructions and several examples of the truth/lie task, after which brain stimulation started and the participants (e) performed a number of practice trials^4^. In all, practice took about 5 min. After that, the participants (f) performed the experimental truth/lie task while tDCS was delivered. Therefore, all participants started performing the experimental task after having been stimulated for at least 5 min. Also, they all finished the task before the stimulation period was over, even though the precise time depended on each participant’s speed of reading aloud and recalling the words. After the truth/lie task, the participants (g) carried out an irrelevant filler task to ensure 20 min of stimulation, since they all were to be assessed in the context of a different study at the end of the experimental session. Finally, they (h) filled in a post-experimental questionnaire asking them whether they experienced any adverse effect during tDCS.

**FIGURE 2:**
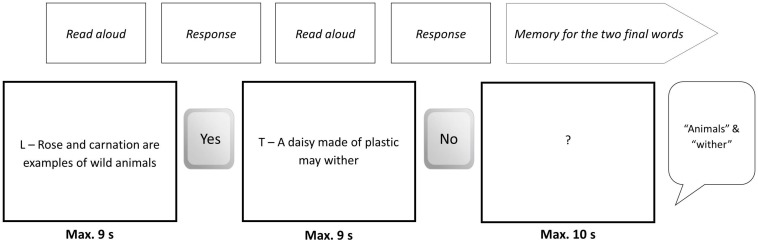
Example of a series of events for a block from Level 2.

The experimental truth/lie task consisted of reading each sentence being displayed on a computer screen aloud and indicating (truthfully or deceptively) whether the statement was true (by pressing a key with a sticker with the word “yes” written on it) or not (by pressing a key with the word “no”). See Figure 2 for an example. At the same time, the participant had to memorize the last word in each sentence. We orthogonally manipulated truthfulness, cognitive load, and brain stimulation.

#### Truthfulness Manipulation

The participants had to reply truthfully to one half of the sentences and deceptively to the other half. The instruction to lie or tell the truth was provided by placing the letter V (“verdad” = “truth”) or M (“mentira” = “lie”) at the beginning of each sentence. We counter-balanced a number of variables. First, one half of the time, telling the truth required saying “no,” while the other half, it required saying “yes.” The same was true for lying. Second, within each level of cognitive load (see below), the order of lying vs. truth telling was randomized. Third, to control for any effect of the specific kind of response required (i.e., deceptive or honest) for each sentence, we built two versions of the entire task counter-balancing the instructions to lie vs. tell the truth.

#### Cognitive Load Manipulation

**FIGURE 3:**
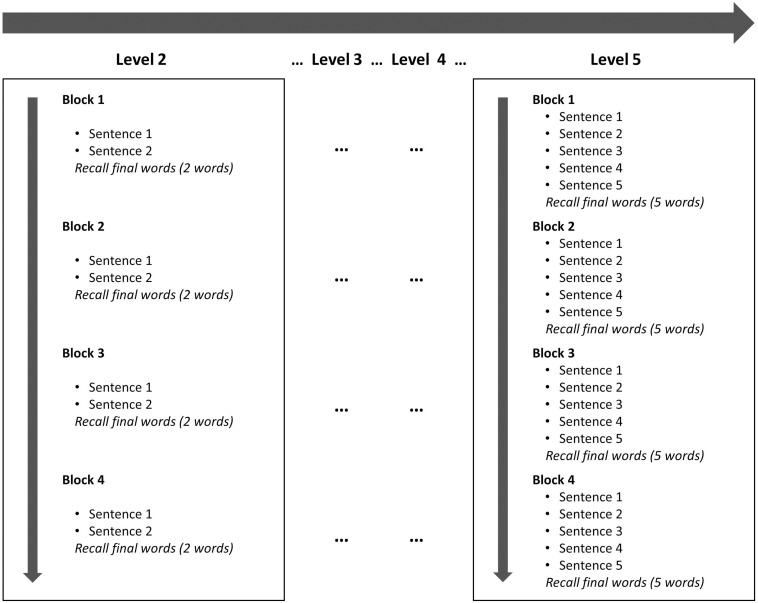
Schematic representation of the load levels.

The sentences were arranged in four levels that were designed to increase cognitive load in a progressive manner. We called them Levels 2, 3, 4, and 5 (there was no Level 1). All participants went from Level 2 through 5 in progression. Each level contained four blocks, and the number of sentences within each block depended on the level. Thus, each block in Level 2 had two sentences, each block in Level 3 had three sentences, and so forth (Figure 3). Therefore, at the end of a Level 2 block (two sentences), the participants had to recall two words (the last word in each of the two sentences presented); at the end of a Level 3 block, the participants had to recall three words; etc^5^. The participants had to say the words aloud, and their replies were audio-recorded. Each sentence was presented in the center of the screen for 9 s, but the time to reply depended upon the number of sentences in the block. Thus, the participants had up to 10 s to reply in Level 2, up to 15 s in Level 3, 20 s in Level 4, and 25 s in Level 5.

#### Transcranial Direct Current Stimulation

The tDCS was delivered through a battery-driven stimulator (neuroConn DC-STIMULATOR) with two saline-soaked surface sponge electrodes (35 cm^2^). The area of interest was the IFC bilaterally. The electrode of interest was placed, depending on the group, either over the right IFC (between F8 and FC6) or the left IFC (between F7 and FC5) according to the 10-10 EEG international system (Jurcak et al., 2007). To minimize its effect on the brain, the reference electrode was always placed extracephalically, over the contralateral shoulder (Ganis, 2014). The combination of both stimulation areas and both electrodes with different polarity (anode and cathode) results in four stimulation conditions: (a) anode over F8–FC6 and extracephalic cathode (hereafter A-F8), (b) cathode over F8–FC6 and extracephalic anode (C-F8), (c) anode over F7–FC5 and extracephalic cathode (A-F7), and (d) cathode over F7–FC5 and extracephalic anode (C-F7). For active stimulation, participants received a constant current of 2 mA intensity for 20 min that faded in and out with an 8 s ramp. For sham stimulation, electrodes were placed at the same position as for the “a” stimulation condition described above, but stimulation lasted for only 30 s.

#### Post-experimental Questionnaire

Finally, the participants completed a questionnaire assessing whether tDCS had any adverse effect on them (Brunoni et al., 2011). None of them reported major complaints or serious discomfort associated with stimulation.

### Data Analyses

The five directional hypotheses were tested with the sham condition data. To test Hypothesis 1, we conducted a one-way, repeated-measure analysis of variance (ANOVA) where cognitive load (Levels 2, 3, 4, 5) was entered as the independent variable and the percentage of words recalled as the dependent measure. Hypotheses 2 and 4 were tested with a cognitive load (Levels 2, 3, 4, 5) × veracity (truthful trials vs. deceptive trials) ANOVA on compliance, whereas Hypotheses 3 and 5 were tested with a similar ANOVA on RT. Wherever the effect of load was statistically significant, we conducted trend analyses to examine whether the data fitted better a linear or a quadratic trend. Also, because the load × veracity interaction effect on compliance approached significance (see below), we conducted additional follow-up analyses comparing truthful vs. deceptive trials separately for each load level and conducted two separate ANOVAs to examine the load effect separately for truthful and deceptive trials.

To explore whether tDCS over the IFC had an impact on memory performance, we ran a mixed ANOVA with stimulation condition (A-F7, A-F8, C-F7, C-F8, sham) as the between-participants factor, load (Levels 2, 3, 4, 5) as the repeated-measure factor, and percentage of words recalled as the dependent variable. Again, trend analyses were conducted for the load main effect.

Finally, the potential impact of brain stimulation on performance during truthful and deceptive trials was examined with two mixed ANOVAs (one on compliance and one on RT). Stimulation condition was entered as the between-participants factor and veracity as the repeated-measure variable. In reality, these two ANOVAs were conducted twice: first, for those load levels where the individual participant was able to recall the whole set of final words in at least 50% of the blocks (efficient condition), and second, for those load levels where the participant was not able to do so (overloaded condition). This was done to take individual differences in working memory into account. Also, to increase statistical power and based on visual inspection of the pattern of the data, in some cases, we conducted additional ANOVAs with only some of the stimulation conditions and compared truthful and deceptive trials within specific stimulation conditions (see section “Results” for details).

## Results

We first examined the data to identify both extreme values [i.e., those deviating more than 3.0 IQR (interquartile range) from the box in the box plot] and atypical values (i.e., those with a 1.5- to 3.0-IQR deviation) for each variable in each condition. The extreme values in either dependent variable were excluded from analyses (Tukey, 1977). In addition, extreme and atypical values in compliance were used to identify those participants who probably had not understood the instructions (and whose data were thus unreliable) to dismiss them altogether. Specifically, we excluded from all analyses those participants with extreme and/or atypical values in compliance in at least three out of the four cognitive load levels^6^. Finally, one additional participant made errors in 40% of the experimental sentences (specifically, she frequently failed to read the sentences aloud and sometimes did not reply within the established time period). Therefore, we assumed she did not understand the instructions, and we dismissed her data. The total number of participants in each condition, along with some descriptive data, is displayed in Table 1. The number of participants included in specific analyses is shown in Tables 2, 3 (these data are available at Open Science Framework)^7^ ^,8^. There were no significant age or digit span differences across stimulation conditions (all *F*s < 1).

**TABLE 2:** Means (Standard Deviations) for recall, compliance, and response time under different levels of cognitive load in the sham condition.

		Compliance (*N* = 16)			Response Time (*N* = 23)		
Cognitive	Recall	Across all	Truthful	Deceptive	Across all	Truthful	Deceptive
Load	(*N* = 24)	trials	trials	trials	trials	trials	trials
Level 2	86.90(10.74)	90.63(8.54)	100.00(0.00)	81.25(17.08)	1.06(0.45)	1.02(0.46)	1.10(0.54)
Level 3	68.75(14.38)	95.25(5.27)	100.00(0.00)	90.50(10.55)	1.23(0.57)	1.06(0.55)	1.39(0.63)
Level 4	51.04(15.27)	87.34(12.51)	93.19(9.97)	81.50(20.29)	1.14(0.51)	1.06(0.59)	1.23(0.52)
Level 5	42.17(10.82)	93.03(5.50)	94.88(6.65)	91.19(8.86)	1.10(0.43)	1.02(0.43)	1.18(0.48)
*Overall*	*62.22 (11.00)*	*91.56 (4.68)*	*97.02 (3.38)*	*86.11 (7.17)*	*1.13 (0.46)*	*1.04 (0.45)*	*1.22 (0.49)*

*Recall and compliance were measured as percentages, and response time in seconds. For compliance, sample size is small because those participants who committed many errors were excluded from analyses (see beginning of section “Results” in text). Because of a technical problem, response time could not be measured for one participant.*

**TABLE 3:** Means (Standard Deviations) for compliance and response time as a function of stimulation conditions in the efficient condition.

	Compliance				Response Time			
Stimulation		Across all	Truthful	Deceptive		Across all	Truthful	Deceptive
Condition	*n*	trials	trials	trials	*n*	trials	trials	trials
A-F7	17	91.99(13.08)	100.00(0.00)	83.97(26.15)	22	1.11(0.47)	0.97(0.43)	1.24(0.58)
A-F8	21	90.68(11.36)	90.48(18.17)	90.87(12.82)	21	1.05(0.37)	1.02(0.39)	1.09(0.41)
C-F7	16	94.71(8.93)	100.00(0.00)	89.41(17.86)	20	1.19(0.36)	1.04(0.39)	1.34(0.46)
C-F8	20	95.21(7.77)	100.00(0.00)	90.42(15.54)	20	1.04(0.42)	0.93(0.37)	1.15(0.56)
Sham	20	94.69(8.29)	100.00(0.00)	89.38(16.58)	22	1.12(0.47)	1.04(0.47)	1.20(0.54)
*Overall*	*94*	*93.42 (9.99)*	*97.87 (9.32)*	*88.96 (17.77)*	*105*	*1.10 (0.42)*	*1.00 (0.40)*	*1.20 (0.51)*

*Compliance was measured as a percentage, and response time in seconds. A-F7, anode over F7–FC5 (left inferior frontal cortex) and extracephalic cathode; A-F8, anode over F8–FC6 (right inferior frontal cortex) and extracephalic cathode; C-F7, cathode over F7–FC5 (left inferior frontal cortex) and extracephalic anode; C-F8, cathode over F8–FC6 (right inferior frontal cortex) and extracephalic anode.*

Hypotheses 1 through 5 were tested for the sham condition. We first describe the analyses testing these hypotheses. After that, we report analyses with all five stimulation conditions to explore whether tDCS modulated the potential effects of load and veracity on performance^9^. Because load and veracity were entered as a factor in some of these analyses, we also indicate whether their effects across all stimulation conditions are consistent with Hypotheses 1, 2, and 3.

### Cognitive Load and Veracity Effects (Sham Condition)

#### Recall

The repeated-measure ANOVA testing of the effect of cognitive load (Levels 2, 3, 4, 5) on memory performance (recall percentage) revealed a reliable effect of load, *F*(3,69) = 150.31, *p* < 0.001, *η^2^_*p*_* = 0.87. Trend analyses showed both the quadratic, *F*(1,23) = 8.62, *p* = 0.007, *η^2^_*p*_* = 0.273, and the linear components, *F*(1,23) = 470.27, *p* < 0.001, *η^2^_*p*_* = 0.95, to be statistically significant, even though the latter accounted for 98% of the variance. These effects indicate that, in line with Hypothesis 1, as cognitive load increased, recall percentage decreased (Table 2).

In a further attempt to ascertain whether our procedure tapped into working memory demands, we correlated the sham participants’ digit span scores with their recall performance in the experimental task. To do so, we calculated an equivalent score (sentence memory span, SMS hereinafter) for the experimental task. More specifically, we computed the highest load level wherein each individual participant was able to correctly recall the whole set of final words in at least two different blocks (out of four). Thus, for example, if a given participant recalled all the words from two blocks for Levels 2 and 3 but not for Levels 4 and 5, his/her SMS was 3. A Pearson correlation analysis showed a reliable association between backward digit span scores and SMS, *r* = 0.56, *p* = 0.004.

#### Compliance

To test Hypotheses 2 and 4, we conducted a cognitive load (Levels 2, 3, 4, 5) × veracity (truthful vs. deceptive response) ANOVA on compliance. The cognitive load main effect failed to reach statistical significance, *F*(2.48,37.14) = 2.76, *p* = 0.066, *η^2^_*p*_* = 0.16 (Huynh–Feldt correction), and pairwise comparisons failed to reveal any significant difference (all *p*s ≥ 0.179). Of note is that compliance was quite high across all load levels (see Table 2). The main effect of veracity was very large and reliable, *F*(1,15) = 50.04, *p* < 0.001, *d* = 1.75, revealing that, as predicted in Hypothesis 2, participants in the sham group were less compliant on deceptive trials than they were on truthful trials (Table 2). Finally, the load × veracity interaction effect was marginally significant, with a relatively large effect size, *F*(3,45) = 2.41, *p* = 0.079, *η^2^_*p*_* = 0.14. Because of its relevance for the present research, we first followed up the interaction by looking at the effect of veracity in each individual load level. Simple effect analyses showed reliable differences between truthful and deceptive trials in Levels 2 (*p* < 0.001, *d* = 1.47), 3 (*p* = 0.003, *d* = 1.15), and 4 (*p* = 0.033, *d* = 0.70), but not 5 (*p* = 0.206, *d* = 0.47). Hence, the magnitude of the veracity effect decreased progressively as load increased, becoming statistically non-significant in the condition with the highest memory load.

Because the lack of a difference in Level 5 seemed to be largely due to performance deflation on truthful trials (see Table 2), we examined the effect of load separately for deceptive and truthful trials. Whereas load level did not affect compliance on deceptive trials, *F*(3,45) = 2.09, *p* = 0.114, *η^2^_*p*_* = 0.12, for truthful trials, the effect was statistically significant, *F*(3,45) = 6.05, *p* = 0.001, *η^2^_*p*_* = 0.29. Specifically, on truthful trials, the participants complied 100% of the time under load Levels 2 and 3; however, their mean compliance across Levels 4 and 5 was *M* = 94.03 (*SD* = 6.75). This figure was statistically lower than compliance across Levels 2 and 3, *F*(1,15) = 12.50, *p* = 0.003, *d* = 0.69. This effect indicates that the increase in cognitive load slightly (but significantly) hindered performance on truthful but not deceptive trials, which clearly goes against our Hypothesis 4.

#### Response Time

To test Hypotheses 3 and 5, we conducted a cognitive load × veracity ANOVA on RT. The cognitive load main effect was only marginally significant, *F*(3,66) = 2.52, *p* = 0.065, *η^2^_*p*_* = 0.10, but pairwise comparisons failed to reveal any significant difference (all *p*s ≥ 0.115). The main effect of veracity was statistically significant, *F*(1,22) = 19.06, *p* < 0.001, *d* = 0.39, revealing that, as predicted in Hypothesis 3, participants in the sham group responded faster to truthful than to deceptive trials (Table 2). The interaction effect did not reach statistical significance, *F*(2.20,48.49) = 1.76, *p* = 0.180, *η^2^_*p*_* = 0.07 (Huynh–Feldt correction). Thus, Hypothesis 5 was not supported: Response latencies for deceptive trials (relative to truthful trials) were similarly longer regardless of load level.

### Effects of tDCS Over IFC on Memory Performance

While our main interest was to examine the effect of tDCS on deceptive behavior, we first looked at its potential modulation of memory performance. Thus, we first analyzed whether SMS varied as a function of the tDCS condition. A one-way ANOVA with stimulation as the factor (A-F7, A-F8, C-F7, C-F8, sham) revealed that this was not the case, *F*(4,108) < 1, *p* = 0.92, *η^2^_*p*_* < 0.01. We also conducted a mixed ANOVA with stimulation (between-group) and load (within-participants) as the factors on the percentages of words recalled. In line with Hypothesis 1 and the above results for the sham condition only, the analysis showed a reliable main effect of load, *F*(3,312) = 475.66, *p* < 0.001, *η^2^_*p*_* = 0.82, and trend analyses showed that although both the linear and the quadratic trend were reliable, the data fitted much better a linear trend, *F*(1,104) = 1163.73, *p* < 0.001, *η^2^_*p*_* = 0.92, than a quadratic one, *F*(1,104) = 57.72, *p* < 0.001, *η^2^_*p*_* = 0.36. However, neither the stimulation effect, *F*(4,104) < 1, *p* = 0.905, *η^2^_*p*_* = 0.01, nor the interaction effect, *F*(12,312) < 1, *p* = 0.866, *η^2^_*p*_* = 0.02, reached statistical significance. These outcomes show that tDCS had no influence on memory performance.

### Effects of tDCS Over IFC on Compliance and RTs During Truthful and Deceptive Trials

The possible effect of tDCS on deceptive behavior was examined considering individual differences in working memory. Specifically, we clustered performance according to the SMS of each participant, so that load was reduced from four to two levels as a function of whether it concerned load conditions under/equal to or over the participant’s SMS (efficient condition or overloaded condition, respectively). Thus, for example, for a given participant with SMS = 3, compliance would be analyzed by averaging performance in load Levels 2 and 3 to comprise the efficient condition, and performance in load Levels 4 and 5 to embrace the overloaded condition. Below we report separate analyses for efficient and overloaded conditions.

#### Efficient Condition

We conducted a mixed ANOVA on compliance with stimulation condition (A-F7, A-F8, C-F7, C-F8, sham) as the between-participants factor and veracity (truthful trials vs. deceptive trials) as the repeated-measures variable. Consistent with Hypothesis 2 and the above results for the sham condition only, the main effect of veracity was statistically significant, *F*(1,89) = 20.39, *p* < 0.001, *d* = 0.63, so that participants were more compliant when responding to truthful than deceptive trials (Table 3). But the omnibus analysis failed to show any reliable effect of either stimulation condition, *F*(4,89) < 1, *p* = 0.539 *η^2^_*p*_* = 0.03, or the interaction, *F*(4,89) = 1.78, *p* = 0.141, *η^2^_*p*_* = 0.07. However, because visual exploration of the data suggested that there were changes in the two conditions involving anodal stimulation, to increase statistical power, we conducted the ANOVA entering only the sham, the anodal left IFC (A-F7), and the anodal right IFC (A-F8) stimulation conditions. This analysis showed that the stimulation condition × veracity interaction approached statistical significance, *F*(2,55) = 2.91, *p* = 0.06, *η^2^_*p*_* = 0.10, and we performed follow-up analyses that confirmed a reliable effect of veracity for the sham (*p* = 0.032, *d* = 0.97) and the left IFC conditions (*p* = 0.003, *d* = 0.93), but not for the right IFC condition (*p* = 0.933, *d* = −0.03). In this latter group, compliance during truthful trials dropped off to the level of deceptive trials (see Table 3). Hence, anodal tDCS over the right IFC hindered performance only when participants were to tell the truth.

The stimulation condition × veracity ANOVA on RT showed the main effect of veracity to be reliable, *F*(1,100) = 28.58, *p* < 0.001, *d* = 0.43. As predicted in Hypothesis 3, deceptive responses required longer times than truthful responses (see Table 3). However, neither the stimulation condition, *F*(4,100) < 1, *p* = 0.813, *η^2^_*p*_* = 0.02, nor the interaction, *F*(4,100) = 1.08, *p* = 0.372, *η^2^_*p*_* = 0.04, was statistically significant. To increase statistical power, and based on visual inspection of the data pattern, we conducted a new analysis including only the Sham, the cathodal left IFC, and the anodal right IFC conditions in the factorial ANOVA. Again, neither stimulation condition, *F*(2,60) < 1, *p* = 0.561, *η^2^_*p*_* = 0.02, nor the interaction, *F*(2,60) = 1.83, *p* = 0.170, *η^2^_*p*_* = 0.06, reached statistical significance, though the veracity effect remained reliable, *F*(1,60) = 14.15, *p* < 0.001, *d* = 0.39.

#### Overloaded Condition

The mixed 5 (stimulation condition) × 2 (veracity) ANOVA on compliance showed that the only reliable source of variability was veracity, *F*(1,108) = 34.45, *p* < 0.001, *d* = 0.69 [for stimulation condition: *F*(4,108) = 1.32, *p* = 0.268, *η^2^_*p*_* = 0.05; for the interaction: *F*(4,108) < 1, *p* = 0.868, *η^2^_*p*_* = 0.01]. Again, the participants were significantly more compliant when responding truthfully (*M* = 92.88, *SD* = 7.41) than deceptively (*M* = 86.67, *SD* = 10.15). A similar ANOVA on response latencies also showed that the only reliable effect was veracity, *F*(1,107) = 48.87, *p* < 0.001, *d* = 0.34 [for stimulation condition: *F*(4,107) < 1, *p* = 0.996, *η^2^_*p*_* = 0.00; for the interaction: *F*(4,107) = 1.58, *p* = 0.186 *η^2^_*p*_* = 0.06]. Deceptive responses required longer times (*M* = 1.24, *SD* = 0.43) than truthful responses (*M* = 1.09, *SD* = 0.44).

## Discussion

Lying has been traditionally considered cognitively more taxing than truth telling, and it is assumed that working memory and inhibitory control play a role in deceptive behavior. Because of the cognitive cost of lying, if cognitive load is artificially increased further (e.g., with a secondary task), the liar’s cognitive system might become overloaded. This, in turn, can produce behavioral indicators that might suggest deception (Vrij et al., 2016, 2017). However, in some circumstances, the level of imposed load can be so high that it can impair not only the liars’ performance but also that of the truth tellers. By progressively regulating the level of induced load, the amount of it hampering the liars’ performance but still not interfering with the truth tellers’ performance could be determined. Therefore, the main goal of this research was to develop a veracity task with an embedded concurrent task to increase cognitive load in a progressive manner, so that we can examine its influence on truthful and deceptive responding. As complementary goals, and because the left prefrontal cortex plays a role in verbal working memory (e.g., Bunge et al., 2000; Owen et al., 2005), and its right counterpart is more clearly involved in inhibitory control (e.g., Anderson and Hanslmayr, 2014; Aron et al., 2014), we wanted to explore whether online stimulation over either the left or the right IFC had an impact on deceptive behavior.

### Cognitive Load and Veracity

The influence of cognitive load on recall, compliance, and RT during deceptive and truthful responding was examined with the sham condition. The analyses revealed that our experimental task was effective in parametrically increasing load: First, the correlation between the participants’ digit span scores and their recall performance in the task (operationalized as SMS) was substantial and reliable. Second, and in line with this finding, we observed that the higher the load level, the lower the participants’ recall (measured as the percentage of words recalled). Importantly, this effect was reliable not only for the sham condition but also across all stimulation conditions. Altogether, these findings indicate that our task imposed actual working memory demands that entitle us to look into the effects of cognitive load on compliance and RT in deceptive and truthful trials.

Our experimental task also worked fairly well to show veracity effects. Specifically, deceptive responding consistently produced both less compliance and longer RTs compared to truthful responding. These effects were very robust and constitute further evidence that lying (even of the simple kind used in our paradigm) recruits more cognitive resources than truth telling. The effect on latencies is consistent with the main conclusions of two recent meta-analyses showing that RT (measured using precise, computer-based paradigms) was longer when responding deceptively than when responding truthfully (Suchotzki et al., 2017; Verschuere et al., 2018).

Thorough examination of performance in the sham condition also revealed that, contrary to our expectation, higher load levels did not produce progressively larger differences between liars and truth tellers. On the contrary, fine-tuned analyses revealed that increasing cognitive load decreased compliance on truthful but not on deceptive trials, such that in Level 5, the difference between truths and lies was no longer significant and had the smallest effect size. This finding underscores the risk mentioned in the introduction that particularly high levels of induced load can be detrimental for truth tellers (e.g., Blandón-Gitlin et al., 2014). Empirical evidence supporting this peril is accumulating: A meta-analysis of 21 individual studies showed that imposing cognitive load does not increase RT differences between truthful and deceptive responses; instead, a small but significant effect was found showing that the induced load decreased the difference by increasing the RT in responding truthfully (Verschuere et al., 2018). This effect might have important consequences if long reaction times (or other cognitive load indicators) are used in applied settings to identify liars, as it can increase the risk of false positives.

In the current experiment, truthful and deceptive trials differed in compliance and RT even in low-cognitive-load conditions. This may suggest that the secondary task *per se* induced sufficient load and that increasing load further (up to Level 5) did not increase the truth–lie differences by decreasing the liars’ performance further. However, as we had no Level 0 condition (a no-load condition wherein the participants would not need to recall any word after each block), we cannot know for sure whether this is the case or, rather, differences between truthful and deceptive trials would have emerged also with no load inducement. Future research should examine this issue.

### Transcranial Direct Current Stimulation

Only a few studies have examined the impact of tDCS of prefrontal regions on deceptive responding, yielding contradictory results and negligible effects (see Bell and DeWall, 2018). Importantly, studies that have used non-invasive brain stimulation techniques others than tDCS also failed to modulate deception. For example, Verschuere et al. (2012) employed continuous theta-burst stimulation (cTBS) to disrupt neural activity in the right inferior frontal sulcus. Their participants were to respond truthfully and deceptively to a set of autobiographical questions. Contrary to their predictions, cTBS had no effect on either RTs or error rates compared to the sham condition.

The heterogeneity of findings across studies can be a consequence of the diversity of procedures and measures used in extant research. This diversity, coupled with the scarcity of studies, makes it hard to draw any firm conclusion and suggests that more research is needed. Hence, an additional goal of the present study was to examine whether tDCS of the left or right IFC (a prefrontal region that has been systematically linked to executive control and deception) had an influence on compliance and RT during deceptive and truthful trials. Our general prediction was that if the IFC plays a role in deceptive behavior (i.e., underpinning working memory and inhibitory control processes), changing neural activity within the ventrolateral prefrontal cortex by means of tDCS should modulate performance in deceptive trials as compared to sham. Importantly, we assessed such a possible neuromodulation effect separately for those load levels wherein participants were not cognitively burdened (efficient condition) and for the remaining load levels (overloaded condition).

While there was no effect of tDCS on compliance or RT in the overloaded condition, a tDCS-related compliance modulation emerged in the efficient condition. Specifically, anodal tDCS of the right IFC rendered truth and lie responses more similar by virtue of disrupting compliance only in truthful trials (all conditions with 100% but A-F8 with 90%). Thus, stimulating the right IFC did not modulate deceptively responding (nor recalling) but affected truthfully responding. This is a striking finding since both deception and activity in the right IFC have been associated with inhibitory control (e.g., Christ et al., 2009; Aron et al., 2014; Walczyk et al., 2014). Hence, in principle, one would expect neuromodulation of this region to impact on the ability to lie rather than tell the truth, which leads us to take the present conspicuous finding with caution and call for further replication with high-powered studies. In addition, some of our analyses were *post hoc* and exploratory; therefore, the outcomes need to be interpreted cautiously. Finally, it is important to note that transcranial stimulation in the present study was mostly delivered while participants were performing the experimental task (online tDCS). It would be interesting to replicate the study with offline tDCS (before the task) to see whether it modulates deceptive behavior and/or interacts with cognitive load when lying.

While the neurocognitive mechanism underlying the observed impairment in responding honestly in the present study is not obvious to us, it does not seem to be related to the active maintenance of task-relevant information in working memory, since none of the stimulation conditions impacted on SMS or the percentage of words recalled. It is important to highlight here, however, that the right IFC is thought to be involved in a number of cognitive functions that include, in addition to deception and inhibitory control, action coordination of multi-component behavior (i.e., Dippel and Beste, 2015; for a functional segregation of the right IFC, see Hartwigsen et al., 2019). Along these lines, a recent study that applied TBS to the right IFC found this region to be causally involved in implementing strategies to organize actions that were to be deployed in cascade (Dippel and Beste, 2015). Hence, and because the experimental task used in the present experiment was necessarily complex in terms of the number and nature of components (i.e., reading and comprehending each sentence, interpreting the truth/lie cue in every trial, maintaining a number of words in working memory, responding accordingly to truthful and deceptive trials, saying the final words aloud…), we speculate that anodal tDCS could have slightly impacted on how the sequence of actions was coordinated, specifically in relation to responding to the truth/lie task. Since it was during truthful trials when participants were more compliant (virtually 100% in our task), performance on these trials had the largest room for being hampered by tDCS. However, while we again acknowledge that any interpretation of our truth-telling-related finding requires caution and further replication, our null result concerning deceptive trials is totally consistent with those from related prior research that used brain stimulation techniques and failed to observe reliable effects (Verschuere et al., 2012; Bell and DeWall, 2018).

### Limitations

Two limitations of the present study deserve to be mentioned. First, the truth/lie task used here may have hindered the modulation of inhibition-related neural activity in the IFC. Indeed, the effect of tDCS over a specific brain region (most likely as a node within a distributed network) is thought to critically depend on the neural engagement of the stimulated region during the cognitive task (Silvanto et al., 2008; Bikson et al., 2013; Miniussi et al., 2013; Rodrigues de Almeida et al., 2019). Hence, while our experimental task worked fairly well by inducing cognitive load and veracity effects, given the number of cognitive operations it involved, its demands for inhibitory control may not have been sufficient for tDCS to change inhibition-related neural activity. Future studies on the role of inhibitory control in lying should consider the use of veracity tasks with a higher load of (and greater specificity for) such a process. Second, the unequal number of males and females in our sample limits our conclusions concerning tDCS, since the small number of males precluded the inclusion of gender as a variable in the statistical analyses. A recent study has reported gender-related individual differences in current distribution (Russell et al., 2014), and we recognize that such differences could have blurred the effects of tDCS in our experiment.

### Conclusion

We designed a new experimental task to increase cognitive load gradually while participants are to either tell the truth or lie regarding general-knowledge facts. The task worked fairly well, and we observed the basic expected effects of veracity and working memory load. We did not find, however, any evidence that cognitive load may impact on lying, since load increases did not enhance the difference between truthful and deceptive trials. Instead, very high load levels reduced compliance in truthful trials, making the truth–lie discrimination based on compliance more difficult. Future refinements of the experimental task can be made to increase the sensitivity of the dependent measures to load manipulation and to make the task usable in other (i.e., more natural) contexts.

On the other hand, tDCS of the IFC (either left or right) had no impact on working memory performance and had only relative influence on compliance and RT during truthful and deceptive trials. Only the anodal stimulation of the right IFC decreased compliance in truthful trials, thus hampering the discrimination between truths and lies. Because non-invasive brain stimulation research is in its infancy, additional studies should be conducted to examine its effect on deceptive responding. However, it is important to highlight that our findings join those from previous studies to show small or null effects of non-invasive brain stimulation techniques on deceptive behavior.

Finally, it is remarkable that both cognitive load and tDCS decreased compliance but only in the truthful trials. In fact, the ability to respond deceptively was not modulated at all by any condition. As previously mentioned, high mental load (and maybe also disruption by tDCS of normal neural activity in the right IFC) would seem to be largely damaging to truth telling (Verschuere et al., 2018; see also the arguments by Blandón-Gitlin et al., 2014). Further research is clearly necessary to replicate, extend, and gain understanding of this putative higher vulnerability of truthful conditions to performance impairment.

## Data Availability Statement

The datasets generated for this study are available at https://osf.io/2ax5s/.

## Ethics Statement

The studies involving human participants were reviewed and approved by the Comité de bioética de la Universidad de Jaén. The participants provided their written informed consent to participate in this study.

## Author Contributions

NS, CG-A, and JM developed the research questions and wrote the manuscript. NS and CG-A designed the study, recruited the participants, and performed the analyses. NS collected the data.

## Conflict of Interest

The authors declare that the research was conducted in the absence of any commercial or financial relationships that could be construed as a potential conflict of interest.

## Footnotes

1 Early studies comparing reaction times when lying and telling the truth (e.g., Henke and Eddy, 1909; Marston, 1920) or examining motor responses while concealing involvement in a crime (e.g., Luria, 1932) were based on theoretical considerations connected to emotional rather than cognitive processes.

2 https://examenes.cervantes.es/es/ccse/examen

3 The number of words is smaller than in Elosúa et al.’s (1996) Spanish adaptation of Daneman and Carpenter’s (1980) Reading Span Test. The reason is that, in this experiment, the instructions to lie or tell the truth were going to increase cognitive complexity.

4 Three Level 2 blocks (i.e., blocks containing two sentences, as explained in the text below) were shown to the participants during the task description. Next, the participants practiced with three Level 2 and two Level 3 blocks.

5 Two additional blocks were placed at the beginning of Level 2 (and after the practice trials) to make participants familiar enough with the actual task. The data from these two initial blocks were not included in the analyses.

6 Note that all sentences were very simple general-knowledge phrases to which anybody would respond with no errors under normal circumstances (as empirically shown in the pilot study; see section “Materials and Methods”). Therefore, participants with extreme and/or atypical errors in compliance in three separate load levels had either not understood the instructions or chosen not to follow them.

7 https://osf.io/2ax5s/

8 The number of participants included in the analyses of the overloaded condition is the same as in Table 1 for all conditions except for sham in measuring RT, wherein *n* = 23.

9 Because stimulation time varied across participants, it is possible that participants stimulated for a longer time (a) reported stronger side effects in the post-experimental questionnaire than their peers and (b) experienced a noticeable effect of tDCS on the dependent measures (though this is unlikely because the measures were taken all through the task, not only at the end of the stimulation period). We conducted a series of analyses to examine these issues. First, we found that scores for the most relevant side effects (headache, scalp pain, tingling, itching, burning sensation, and sleepiness) were low and did not change as a function of either tDCS condition or stimulation duration. Second, stimulation duration had no significant effect on either word recall or compliance, though longer stimulation times were associated with longer RTs. This latter effect merely reflects that slow participants (longer RTs) finished the task later than fast participants; therefore, they were stimulated for a longer time. In short, while tDCS duration should be considered in future experiments, it does not seem to have influenced the current pattern of results. All these analyses are available from the corresponding authors on request.
